# Comparison of gluteus medius muscle activity in Haflinger and Noriker horses with polysaccharide storage myopathy

**DOI:** 10.1111/jpn.13504

**Published:** 2021-02-20

**Authors:** Rebeka Roza Zsoldos, Negar Khayatzadeh, Johann Soelkner, Ulrike Schroeder, Caroline Hahn, Theresia Franziska Licka

**Affiliations:** ^1^ Division Livestock Sciences Department of Sustainable Agricultural Systems University of Natural Resources and Life Sciences Vienna Vienna Austria; ^2^ School of Agriculture and Food Sciences The University of Queensland Gatton Qld Australia; ^3^ Department for Companion Animals and Horses University of Veterinary Medicine Vienna Vienna Austria; ^4^ Royal (Dick) School of Veterinary Studies University of Edinburgh Midlothian UK

**Keywords:** Haflinger, muscle activity, Noriker, polysaccharide storage myopathy, surface electromyography

## Abstract

Type 1 polysaccharide storage myopathy caused by genetic mutation in the glycogen synthase 1 gene is present in many breeds including the Noriker and Haflinger horses. In humans, EMG has already been used to document changes in the muscle activity patterns of patients affected by human glycogen storage disorders. Therefore, the aim of the present study was to describe gluteus muscle activity with surface electromyography (sEMG) in Haflinger and Noriker horses with known GYS1 mutation status during walk and trot. Thirty‐two horses (11 Haflinger and 21 Noriker horses) with homozygous non‐affected (GG), heterozygous affected (GA) and homozygous affected (AA) status of GYS1 mutation without overt clinical signs of any myopathy were selected for the current study. Using surface electromyography gluteus medius muscle activity at walk and at trot was measured, and muscle activity was described in relation to the maximum observed value at the same sensor and the same gait. In order to further describe the signals in detail comprising both frequencies and amplitudes, the crossings through the baseline and the 25, 50 and 75 percentile lines were determined. The result of the relative muscle activity did not show a consistent difference between affected and non‐affected horses. Genetically affected (GA and AA) horses showed significantly less density of muscle activity for both gaits and horse breeds except for the crossings per second at the baseline and 75 percentile at walk in the Haflinger horses and 75 percentile at trot in the Noriker horses. The medians of all calculated density values were significantly lower in the GA Haflingers compared to the GG Haflingers (*p* = 0.012) and also in the AA Norikers compared to the GG Norikers (*p* = 0.011). Results indicate that the GYS1 mutation reduces the number of functional muscle fibres detected by sEMG measurements even in the absence of overt clinical signs.

## 1 INTRODUCTION

In the equine gluteus medius muscle (GM), regional fibre variations have been reported (Bruce & Turek, 1985; Grotmol et al., 2002). For aerobic, slower, continuous work (e.g. for joint stabilization) type I fibres are responsible for this activity; these are mostly found in the deeper layer of the muscle. For anaerobic, fast bursts of work such as joint movements during locomotion type II fibres are used, these are located in the superficial layer of the muscle. Such muscle functions can be affected by several equine disorders, such as myopathies creating dysfunctional muscle fibres (McCue et al., 2008). In horses, myopathies may be inherited (such as polysaccharide storage myopathy, PSSM) or acquired (such as rhabdomyolysis) (McCue et al., 2009). In humans, glycogen storage diseases of muscles also exist, and these are caused by mutations in genes controlling enzymes that metabolize glycogen and glucose. These human glycogen storage diseases are in some regards similar to equine PSSM, a muscular defect in horses, which is defined by the irregular build‐up of the normal form of sugar stored in muscle (glycogen) as well as an irregular form of sugar (amylase‐resistant polysaccharide) in muscle tissue (Valberg et al., 2011). Two types of PSSM are differentiated: type 1 PSSM is the form of PSSM caused by the genetic mutation in the glycogen synthase 1 gene (GYS1), while type 2 PSSM is characterized by abnormal polysaccharide in muscle biopsies without this GYS1 mutation (McCue et al., 2009). Type 1 PSSM is inherited as an autosomal dominant trait, and therefore, only one parent needs to pass it on, with a risk that offspring will develop the clinical disease.

The use of electromyography (EMG) is now widespread for the investigation of questions into equine movement and biomechanics (Kienapfel et al., 2017; St. George & Williams, 2013), and it has also been used to detect neurological deficits (Wijnberg et al., 2004). In humans, EMG has already been used to document changes in the muscle activity patterns of patients affected by human glycogen storage disorders (Bruno et al., 1993; Cornelio et al., 1984; Jeub et al., 2006). Specifically, abnormal electromyograms, described either as short duration and low amplitude motor unit potentials in the proximal muscles or decreased number of motor unit potentials in the proximal and distal muscles, were found in such patients (Cornelio et al., 1984), while in another study positive sharp waves and fibrillation potentials were found to be associated with myopathy (Bruno et al., 1993). More recently, myopathic changes were found to be associated with pathological spontaneous EMG activity and pseudomyotonic discharges (Jeub et al., 2006). Similar EMG changes might be present in horses with PSSM type 1 mutation; however, the degree of clinical disease present with the PSSM1 mutation varies between breeds; probably as gene interactions, diet and exercise vary between individuals and between breeds (Pullman et al., 2000). Even horses without overt clinical signs of PSSM (such as muscle atrophy) show histological changes of muscle fibres, and they therefore may exhibit a different muscle activity pattern compared to horses without the PSSM1 mutation.

The aim of the present study was to compare the surface muscle activity of the equine GM muscle in Haflinger and Noriker horses with known GYS1 mutation status during walk and trot. The following hypotheses were examined: (a) in both breeds, the surface muscle activities show decreased activity in affected horses compared to non‐affected horses and (b) the effect of GYS1 mutation status is larger than the effect of breed on the muscle activity pattern.

## 2 MATERIALS AND METHODS

### 2.1 Animals

Data were collected from two distinct subgroups, from 210 Noriker horses and from 14 Haflinger horses. All these horses were presented as healthy by their owners or handlers and had hair root samples taken from the tail or mane for DNA extraction and genetic analyses and tested for GYS1 mutation. For the present study, 7 Noriker triplets (GG, GA, AA) were selected (21 horses), as well as two similar groups of Haflingers (GG 6 horses, and GA 5 horses) based on the criteria sex, age, body mass. In the present study, measurements obtained in two previous studies were used. A summary of the horses is shown in Table 1.

**Table 1:** Summary of horses used in the study

	Noriker horses (*n* = 21)		Haflinger horses (*n* = 11)
	Mares (*n* = 15)	Stallions (*n* = 5)	Mares (*n* = 11)
Age mean ± *SD*, years	7 ± 3	6 ± 5	7 ± 3
Body mass mean ± *SD*, kg	736 ± 66	670 ± 76	455 ± 44
GG homozygous non‐affected, *n*	5	2	6
GA heterozygous affected, *n*	6	1	5
AA homozygous affected, *n*	5	2	–

Abbreviations: AA, homozygous affected; GA, heterozygous affected; GG, homozygous non‐affected, SD, standard deviation.

#### 2.1.1 Noriker horses assessed in hand

Horses with homozygous non‐affected (GG), heterozygous affected (GA) and homozygous affected (AA) status of GYS1 mutation were used. These horses were presented by the breeders or handlers and measured at walk and trot along a straight line of about 10 m. The study of Noriker horses was approved by the Commission for Ethics and Animal Welfare, University of Veterinary Medicine Vienna; protocol number and ETK‐20/06/2016.

#### 2.1.2 Haflinger horses assessed on the treadmill

Here, only horses with homozygous non‐affected (GG) and heterozygous affected (GA) status of GYS1 mutation were used. These horses had been part of a previous study into the effect of feeding a carbohydrate‐rich diet compared with hay‐only diet muscle enzyme concentrations after treadmill exercise was studied (Schroeder et al., 2015). Haflinger horses were handled by handlers known to them and measured at walk and at trot on a zero‐per cent incline treadmill. They underwent a strict diet and exercise regime. For these horses, superficial muscle biopsies of GM were taken and histological tests were carried out. In these samples, no difference was found regarding fibre size, fibre size variation and fibre type distribution with roughly 30% each of type I, type IIa and type IIb fibres found in both groups. Total myopathy score (including parameters such as anguloid atrophy, necrosis/macrophages and central nuclei) in affected horses was significantly higher than in control horses (*p* = 0.04) (Schroeder et al., 2015). The study of the Haflinger horses was approved by the institutional ethics committee of the University of Veterinary Medicine Vienna and the national authority according to § 8ff of Law for Animal Experiments (Tierversuchsgesetz TVG, GZ 68.205/0169‐II/ 3 b/2012).

### 2.2 Data collection

All horses were measured at their natural speed three times 10 s both at walk and at trot, starting with all horses measured at walk, in order to allow the warm‐up, followed by the trot measurements. A minimum of ten strides was used for further analysis.

#### 2.2.1 Accelerometer and muscle activity measurements

Wireless surface electromyography (Delsys Trigno) sensors with in‐built 3‐axial accelerometers and wireless electrodes (each sensor consisting of four parallel metal bars with an integrated amplifier, size 27 × 37 × 15 mm, mass 14.7 g) were placed bilaterally and parallel to the fibre orientation over the left and right gluteus medius muscle. Due to the two different set‐ups used in the studies from which the measurement were combined in the present study, the locations for sEMG electrodes were tested with (Noriker horses) and without (Haflinger horses) hair. In Noriker horses, the coat was thoroughly cleaned with a brush following by diligent dust removal with a textile towel, while in Haflinger horses the coat was shaved with a razor, and the skin was cleaned using isopropyl alcohol on slightly abrasive, roughly woven swabs. The exact position of the electrode was at the midpoint between origin and insertion in the middle of the widest section of the GM; this was identified by palpation of the tuber coxae, tuber sacrale and 3rd trochanter of the femur. The Delsys Adhesive Sensor Interface and additional adhesive tape were used to position the electrodes on the skin. After electrode placement, and prior to obtaining the sEMG measurements, the Delsys Real‐time Signal Quality Monitor tool was used to check the signal quality and the presence of line interference for each electrode. At walk and at trot surface, electromyography (sEMG) at sampling frequency of 2000 Hz collected the muscle activities and tri‐axial acceleration (ACC) at sampling frequency of 148 Hz collected the accelerations from the hindlimbs.

### 2.3 Data processing

#### 2.3.1 Accelerometer data processing

**Figure 1:**
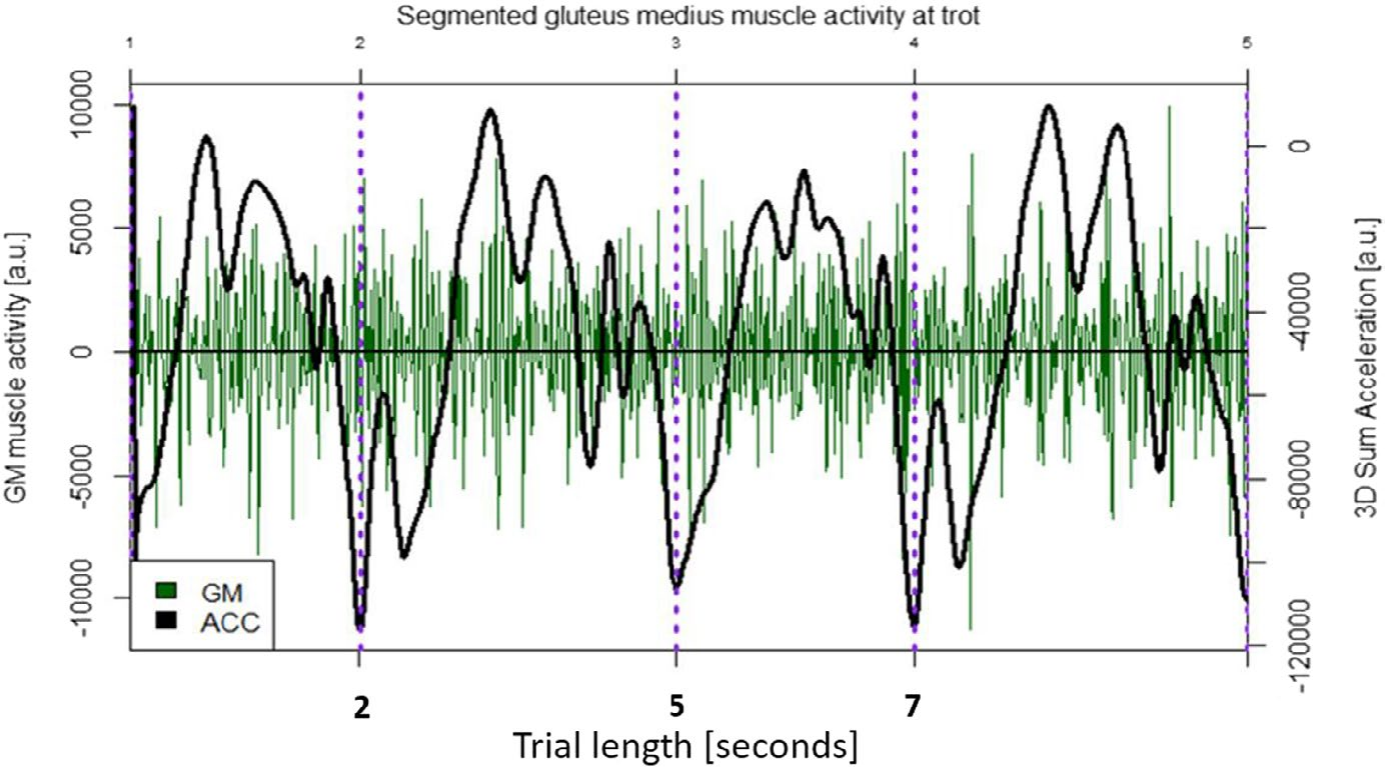
The segmented gluteus medius muscle activity signal (green lines) of a Noriker horse shown with the crossings per seconds at trot (CS/s, i.e. the number of times the signal trace passes a defined value line) together with the accelerometer (ACC) curve of the hindlimb (black lines), vertical lines (purple lines) identifying the segmented motion cycles. A.u.: arbitrary unit. Trial length in seconds: represents the timeline of the measurements

Three‐dimensional (3D) sum acceleration of left and right gluteals were calculated. The acceleration data were resampled to 120 Hz, to fit it to the frequency of the resampled sEMG data. The continuous data were then segmented into motion cycles by cutting at the occurrence of the local minima of the 3D sum acceleration of the left and right hindlimbs, and sEMG data were cut accordingly (Figure 1).

#### 2.3.2 Surface electromyography signal processing

For all the data analysis, values are presented as calculated and in relation to values of the healthy horses of the same breed (GG = 100%). This was done to allow comparison of data obtained in different measurement set‐ups.

#### 2.3.3 Arbitrary composite value

**Figure 2:**
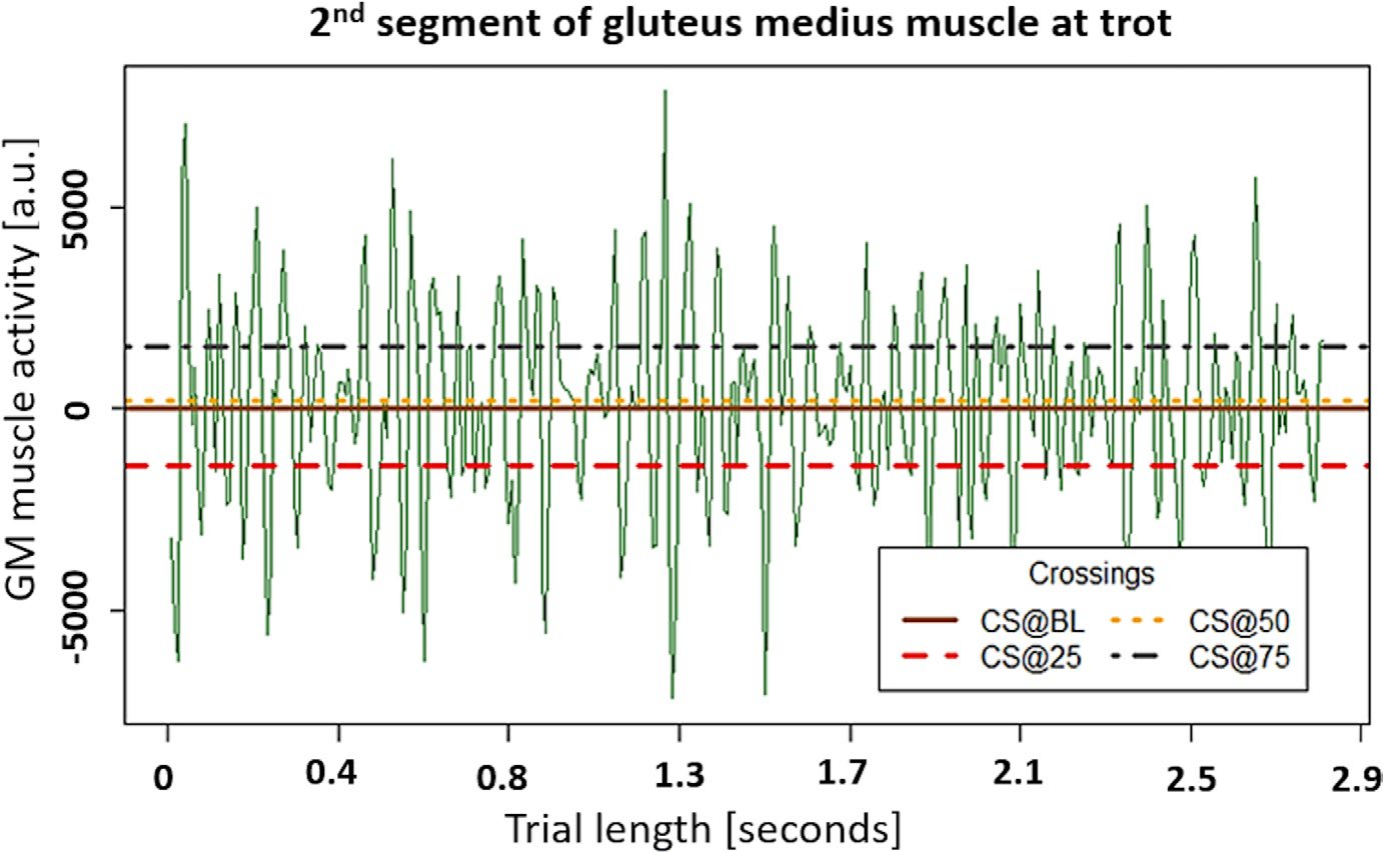
One muscle activity segment selected from the trial of a Noriker horse to show a closer observation of the crossings. With the horizontal lines displayed over gluteus medius muscle activity the crossings (CS) at the baseline (CS/s@BL) was 99, the crossings at the 25 percentile (CS/s@25) was 91, the crossings at the 50 percentile (CS/s@50) and the crossings at the 75 percentile (CS/s@75) was 90. Please note that baseline (CS/s@BL) and the crossings at the 50 percentile (CS/s@50) very close. A.u.: arbitrary unit. Trial length in seconds: represents the timeline of the measurements

Additionally, the sEMG signal was reported as an arbitrary unit, with signals normalized to the range of sEMG values of each sensor in each gait. A novel composite value reflecting the signal frequency at several signal amplitude was determined: incidents where the signal crosses a defined percentile value of the sEMG amplitude, either by exceeding it or by falling below it were expressed as crossings per second (CS/s), that is the frequency of the trace line passing a value in either direction (Figure 2). This newly established composite value is termed ‘density’ in the remainder of the present paper. The density values were based on the sEMG data of each motion cycle and each sensor at the baseline (for CS/s@BL), the 25 percentile (for CS/s@25), the 50 percentile (for CS/s@50) and the 75 percentile (for CS/s@75) (Figure 2).

#### 2.3.4 Maximum muscle activity

After DC offset removal, high‐pass filtering (5th order Butterworth filter with 40 Hz cut‐off), full‐wave rectification and resampling data were normalized to the maximum observed value of the muscle activity measured at each sensor and each gait and expressed as per cent (%MOA).

### 2.4 Statistical analysis

Statistical analyses were done in the statistical program R (Team, 2013) using the MASS package (Venables & Ripley, 2002). Normality of distributions was tested with Shapiro–Wilk tests. For the group comparisons at each density level, non‐parametric data were tested using a Kruskal–Wallis rank sum test and pairwise comparisons between group levels with Bonferroni corrections for multiple testing. Comparison of the medians of all density levels in each breed and genetic group was carried out using Friedman's test with a Bonferroni correction for the Norikers (AA, GA, GG) and Wilcoxon signed‐rank tests for the Haflingers (GA, GG). Pearson correlation coefficient was calculated to express correlations between muscle activities and GYS1 mutation status. In all of the tests above, the level of significance was set at 0.05.

## 3 RESULTS

**Figure 3:**
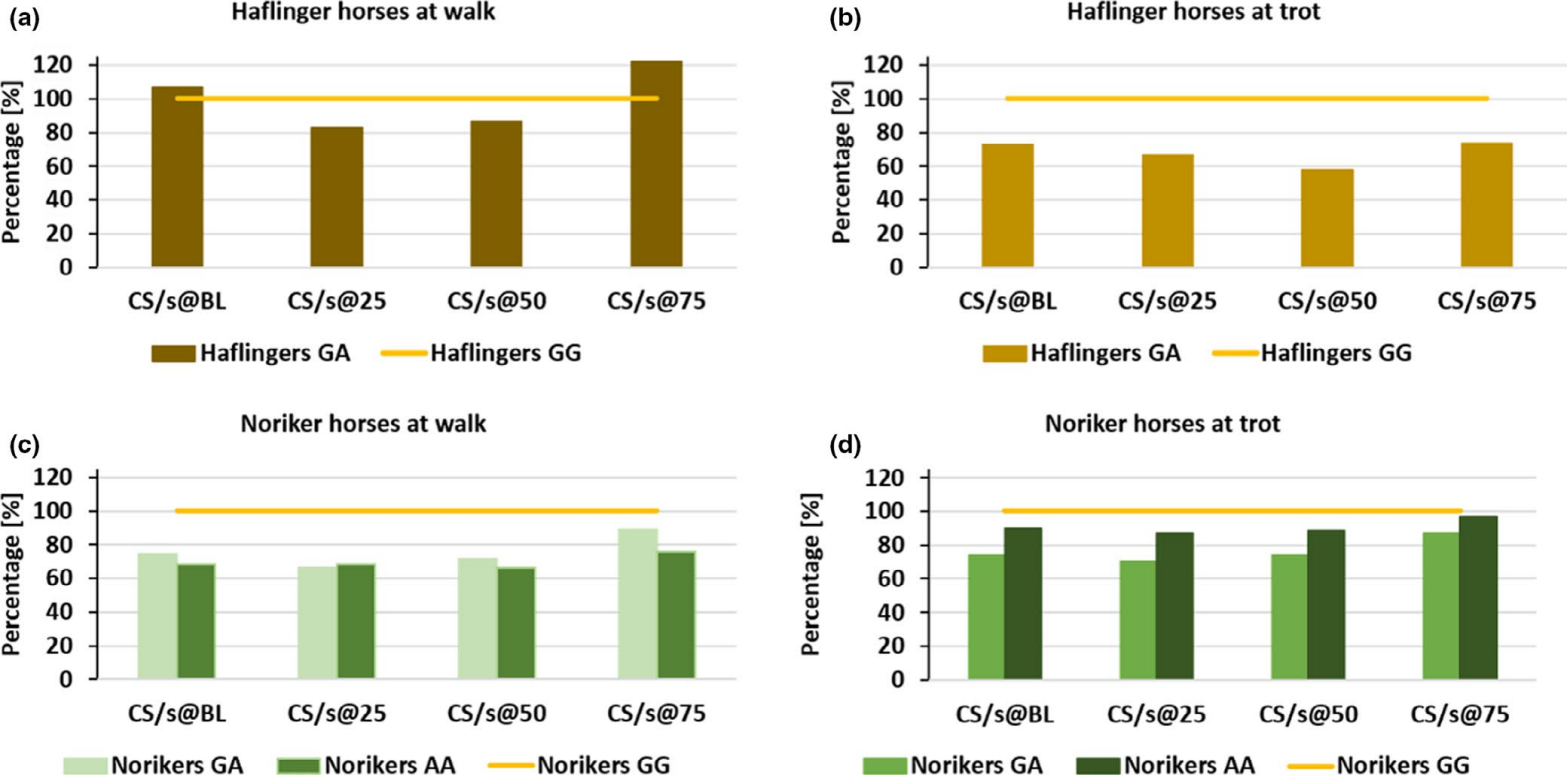
Homozygous non‐affected horses considered as 100%. Relative muscle activity of the affected horses is shown as normalized to the CS/s of non‐affected horses of the same breed at BL, at 25%, 50% and 75% at walk and trot respectively. AA, homozygous affected; GA, heterozygous affected; and GG, homozygous non‐affected

**Figure 4:**
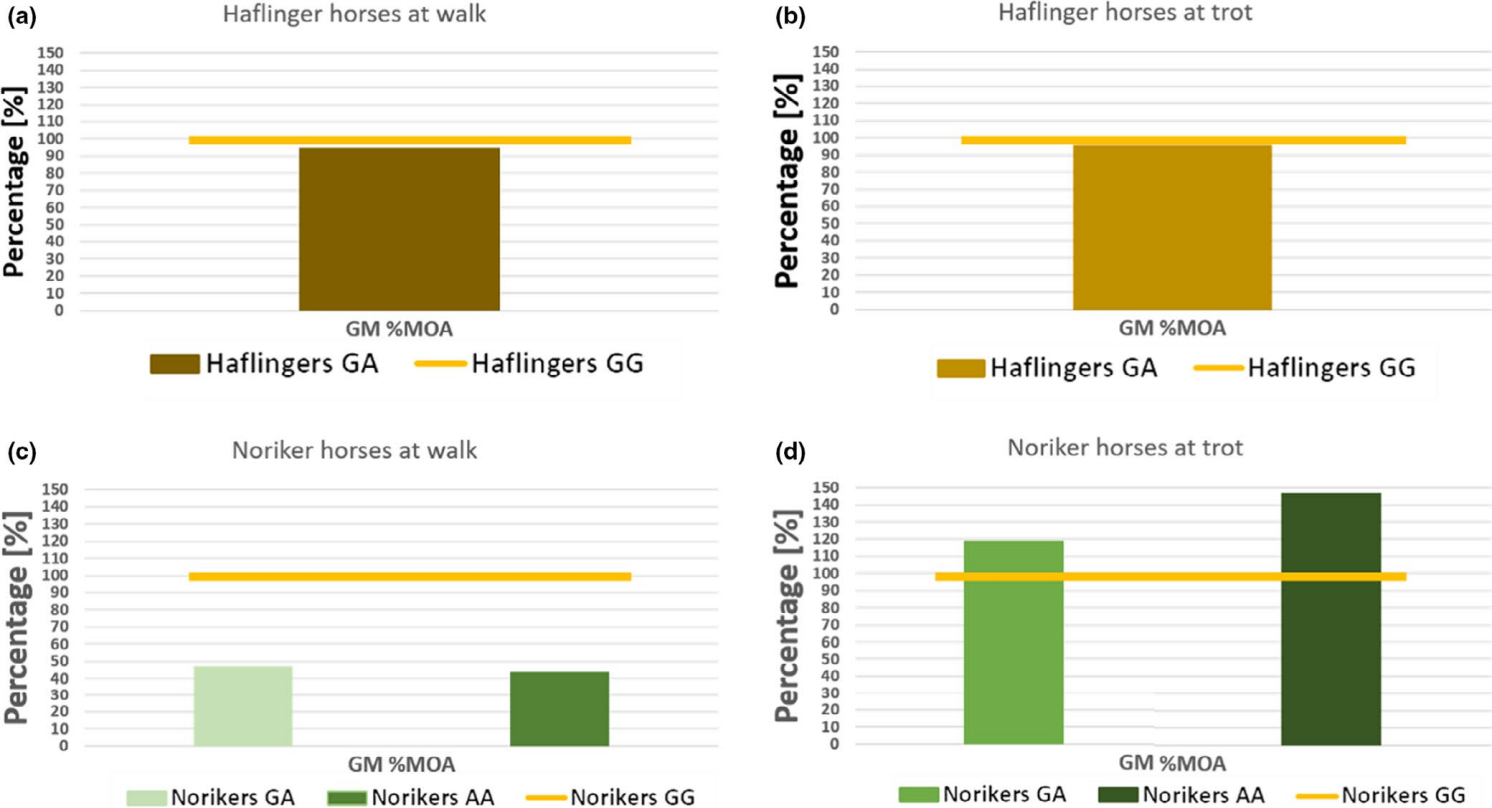
Homozygous non‐affected horses considered as 100%. Relative muscle activity of the affected horses is shown as normalized to the %MOE of non‐affected horses of the same breed at walk and trot respectively. GG: homozygous non‐affected, GA: heterozygous affected, AA: homozygous affected, maximum observed value of the muscle activity (%MOA)

Comparing the results of data in relation to values of the healthy horses of the same breed (GG = 100%), for all horses and for both gaits the muscle activity densities (determined as described above) could be identified (Figures 3a–d and 4a–d). Based on the new composite values in Noriker horses at walk and at trot and in Haflingers at trot, the genetically affected (GA and AA) horses had relative muscle activities below the genetically healthy (GG) horses (Figure 3b–d). Maximum muscle activity (%MOA) of Noriker horses at walk and of Haflingers at walk and trot showed the genetically affected (GA and AA) horses to have a relative muscle activity below the genetically healthy (GG) horses (Figure 4a–c). For both methods, no statistical tests were carried out, as they were only used for normalization purposes to allow the data to be compare between the two different measurement set‐ups.

The median values of the muscle activity densities for CS/s@BL ranged 34–51 with IQRs 30–60 in Noriker horses and 12–16 with IQRs 10–16 in Haflinger horses. The median values of the muscle activity densities for CS/s@25 ranged 24–39 with IQRs 21–44 in Noriker horses and 4–11 with IQRs 2–13 in Haflinger horses. The median values of the muscle activity densities for CS/s@50 ranged 32–50 with IQRs 25–60 in Noriker horses and 8–16 with IQRs 4–16 in Haflinger horses. The median values of the muscle activity densities for CS/s@75 ranged 24–41 with IQRs 23–47 in Noriker horses and 9–12 with IQRs 5–14 in Haflinger horses (Tables 2 and 3).

**Table 2:** Median and interquartile ranges of gluteus medius muscle activity of Haflinger horses walking and trotting on the treadmill

Haflinger horses	GG (*n* = 6)		GA (*n* = 5)	
	Median	IQR	Median	IQR
WALK CS/s@BL	15	13–16	13	11–15
TROT CS/s@BL	16	15–16	12	10–15
WALK CS/s@25	9	7–10	4	2–6
TROT CS/s@25	11	11–13	9	7–9
WALK CS/s@50	15	13–16	8	4–11
TROT CS/s@50	16*	15–16	10* (*p* = 0.032)	6–12
WALK CS/s@75	10	9–12	9	7–12
TROT CS/s@75	12	11–13	9	5–14
WALK %MOA	13	10–15	11	11–12
TROT %MOA	12	11–13	12	10–15
Overall CS/s median comparisons	GG <> GA		*p* = 0.012	

Note

The crossings at the baseline per seconds (CS/s@BL), the crossings at the 25 percentile per seconds (CS/s@25), the crossings at the 50 percentile per seconds (CS/s@50) and the crossings at the 75 percentile per seconds (CS/s@75) and the relative to maximum observed activity (%MOA). Asterisks (*) indicate significant differences between these two values.

Abbreviations: AA, homozygous affected; GA, heterozygous affected; GG, homozygous non‐affected, SD, standard deviation.

**Table 3:** Median and interquartile ranges of gluteus medius muscle activity of Noriker horses walking and trotting in hand

Noriker horses	GG (*n* = 7)		GA (*n* = 7)		AA (*n* = 7)	
	Median	IQR	Median	IQR	Median	IQR
WALK CS/s@BL	45	39–52	34	32–36	35	30–37
TROT CS/s@BL	50	45–60	51	40–56	40	38–53
WALK CS/s@25	37*	29–39	24* *p* = 0.037	21–26	27	24–30
TROT CS/s@25	39	33–44	33	29–39	28	27–37
WALK CS/s@50	42	38–48	32	25–34	29	28–32
TROT CS/s@50	50	44–60	49	37–55	39	31–54
WALK CS/s@75	29	26–33	24	23–27	24	26–26
TROT CS/s@75	41	30–47	34	30–44	34	30–47
WALK %MOA	10	7–14	6	4–7	6	4–8
TROT %MOA	10	8–12	12	8–13	16	11–19
Overall CS/s median comparisons			GG <> AA		*p* = 0.011	

Note

The crossings at the baseline per seconds (CS/s@BL), the crossings at the 25 percentile per seconds (CS/s@25), the crossings at the 50 percentile per seconds (CS/s@50) and the crossings at the 75 percentile per seconds (CS/s@75) and the relative to maximum observed activity (%MOA). Asterisks (*) indicate significant differences between these two values.

Abbreviations: AA, homozygous affected; GA, heterozygous affected; GG, homozygous non‐affected, SD, standard deviation.

The median values of the %MOA for 6–10, with IQRs 4–14 in Norikers at walk and 11–13 with IQRs 10–15 in Haflingers at walk. The median values of the %MOA for 12–16, with IQRs 8–19 in Norikers at trot and 12–13 with IQRs 10–15 in Haflingers at trot (Tables 2 and 3).

The statistical test results of pairwise comparisons between group levels with corrections for multiple testing found the muscle activity densities for CS/s@50 in Haflingers at trot between the different groups of GYS1 mutation status were found to be significant (*p* < 0.04) (Table 2). In Noriker horses, test results of pairwise comparisons between group levels with corrections for multiple testing found the muscle activity densities for CS/s@25 at walk were significantly different between the GG and GA (*p* < 0.04) (Table 3).

Comparison of the medians of all density levels in Norikers showed significant differences between the GG and AA (*p* = 0.011) and in Haflingers between the GG and GA (*p* = 0.012) (Table 3).

Pearson correlation coefficient showed significant correlations with healthier having higher values in CS/s@BL (*r* = −0.52), in CS/s@25 (*r* = −0.46) and in CS/s@50 (*r* = −0.48) at walk in the Noriker horses (*p* < 0.05) and in CS/s@50 (*r* = −0.67) at trot in the Haflinger horses (*p* < 0.04).

**Figure 5:**
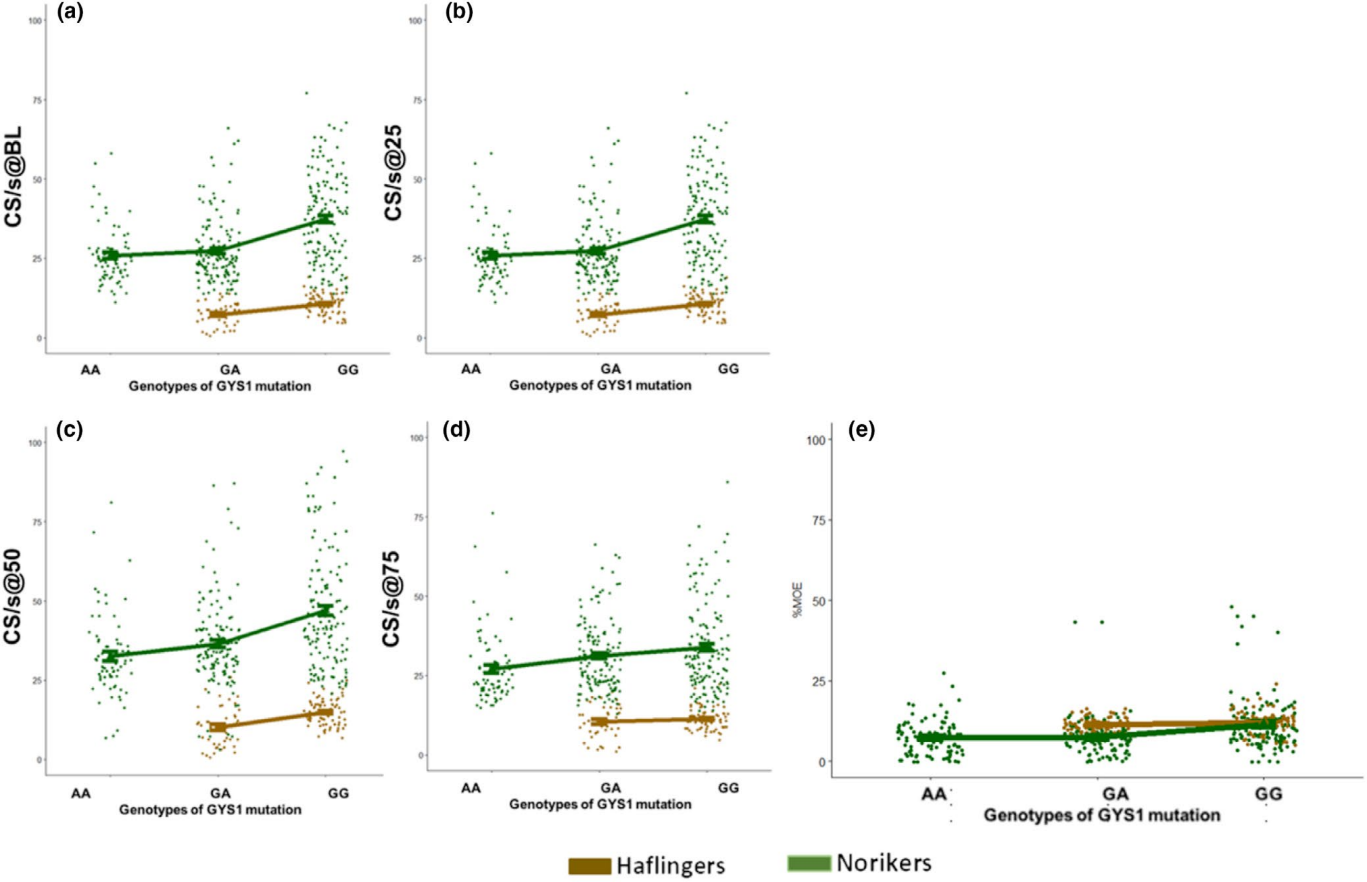
Distribution of density of muscle activity in Haflinger and Noriker horses with GYS1 mutation status of homozygous non‐affected (GG), heterozygous affected (GA) and homozygous affected (AA). Each (non‐normalized) value collected both at walk and at trot is represented as a single dot. (a) Crossings at the baseline per seconds (CS/s@BL), (b) the crossings at the 25 percentile per seconds (CS/s@25), (c) the crossings at the 50 percentile per seconds (CS/s@50) and (d) the crossings at the 75 percentile per seconds (CS/s@75) and (e) the relative to maximum observed activity (%MOA)

The distribution of density of muscle activity in Haflinger and Noriker horses was very similar within the status of GYS1 mutation; however, the Haflingers showed lower values throughout (Figure 5a–d), while the %MOA did not show the genetic difference (Figure 5e).

## 4 DISCUSSION

In the present study, data were analysed using two different approaches, a more traditional analysis documenting the relationship between the maximum observed values measured at each sensor in each gait, which is recommended for normalization (St. George et al., 2019; Zsoldos et al., 2014). This did not show a consistent and convincing difference between affected and non‐affected horses. However, based on the histology of PSSM and the reduced number of functional muscle fibres (Schroeder et al., 2015), in the present paper a novel analysis of the sEMG data was applied. With this, we could show that throughout the locomotion the density of the gluteus medius muscle activity is higher in the majority of the non‐affected horses than in the genetically affected horses; this is true for both walk and trot. Higher values were found for the affected horses for the baseline and for the 75 percentile values at walk in Haflingers and for the 75 percentile values at trot in Noriker horses. The reason for this may be that even in the absence of overt clinical signs of PSSM, such as muscle atrophy, subtle histology signs of myopathy were seen in the GA Haflingers and Norikers, as the myopathy score was significantly higher than in the GG Haflingers (Schroeder et al., 2015). Thus, the mutation seems to lead to a reduction in functional muscle fibres available for the measurement with sEMG.

The electrical activity of the muscle may also be influenced by the fibre types sampled by the electrodes used; however, this is still under investigation. The Haflingers used in the present study did not show differences in their fibre types on biopsy of the gluteus muscle. Also, the depth of the area sampled by sEMG measurement is not clearly defined, and therefore, the difference in fibre types between more superficial and deeper areas of the gluteus muscle (Payne et al., 2005) may have been influential, that should be documented in future using intramuscular EMG readings.

In the different draft horse breeds all across Europe and North America, type 1 PSSM I is present (Baird et al., 2010). Allele age estimations indicating that the underlying GYS1 mutation with the GYS1 Arg309His allele originated 1200–1500 years ago, and many draft horse breeds today were derived from these horses (McCue et al., 2008, Valentine et al., 1999). This is continued to be preserved in many breeds of the domestic horse (McCoy et al., 2014). In the relatively old Austrian Noriker breed and in other older European horse breeds (Belgian Draft and South German Coldblood), the GYS1 Arg309His allele is present, and a significant percentage of founder mares of the Haflinger breed are thought to have been Norikers (Druml et al., 2017, 2008).

The large influence of diet and exercise on the clinical disease of type 1 PSSM has been documented (Baird et al., 2010). In the Haflingers in the present study, management factors such as diet and exercise were strictly controlled, while in the Norikers a large variety of environmental factors were present as neither diet nor exercise had been standardized. Despite these differences, the effect of the GYS1 mutation on the sEMG measurements was similar in both breeds.

In humans, a glycogen debrancher enzyme deficiency disease exists (Cori disease, also known as glycogen storage disease type III). Cornelio et al. (1984) showed in two patients with clinically apparent debrancher deficiency that the EMG profile included short duration and low amplitude motor unit potentials in their proximal muscles and that the motor unit potentials were increased in duration and amplitude in the first dorsal interosseus of the hands and in the tibialis anterior. Such an increased duration of motor unit potentials (i.e. reduced frequency of motor unit potentials) corresponds well with the decreased muscle activity density, also reflecting reduced muscle activity frequency, found in the horses with GYS1 mutation in the present study. In the aforementioned study, there were also abundant fibrillations in every muscle tested, and the number of motor unit potentials was decreased (Cornelio et al., 1984).

For the present study, Noriker and Haflinger horses were selected with similar age ranges and body mass to decrease the body size differences between the two breeds. In the Noriker breed, triplets of horses (AA, GA, GG) could be identified, which is ideal. Unfortunately, this was not possible in the Haflinger horses, where all horses were mares, but ages and body masses did not allow such an exact pairing of individuals, therefore they were analysed as reasonably homogenous groups.

The matching for the age of the animals was especially important, as muscle fibres are still developing in young individuals, leading to MUPs of shorter duration and lower amplitude than in adult or older horses (Wijnberg et al., 2003), which should not be misinterpreted as myopathy. With ageing, the muscle starts to degenerate mildly, resulting in larger, broader MUPs, which again should not be mistaken for mild neuropathy (Wijnberg et al., 2003).

The measurements of the Noriker horses were carried out over ground, while the measurements of the Haflinger horses were carried out on the treadmill and in both cases the results were time normalized. It is well known in the equine biomechanics field that there are major differences to study muscle function over ground or on the treadmill (Buchner et al., 1994), one of the main functional difference could be that the GM muscle function during locomotion is less forceful on the treadmill compared to over ground. Despite these obvious differences in the muscle use required, the present study showed that the muscle results did not differ between the Noriker and Haflinger horses, indicating the stability of the method.

## 5 CONCLUSIONS

Muscle activity relative to the maximum observed value does not show consistent differences between horses with and without mutation in the glycogen synthase 1 gene (GYS1). Using this novel method, the arbitrary composite value of muscle density, such differences can be shown. Specifically, the reduced frequency and reduced amplitude present in the horses with the mutation potentially reflect a reduced number of muscle fibres sampled by each sEMG sensor, even in the absence of overt clinical signs. We believe that this shows the potential of using surface electromyography to document myopathy even without overt clinical signs.

## CONFLICT OF INTEREST

None of the authors of this paper has a financial or personal relationship with other people or organizations that could inappropriately influence or bias the content of the paper.

## ANIMAL WELFARE STATEMENT

The authors confirm that the ethical policies of the journal, as noted on the journal’s author guidelines page, have been adhered to and the appropriate ethical review committee approval has been received. The authors confirm that they have followed EU standards for the protection of animals used for scientific purposes.

## Data Availability

The data that support the findings of this study are available from the corresponding author upon reasonable request.
